# Comparison of Postoperative Pain and Appetite in Pediatric Patients Undergoing Monopolar Tonsillotomy and Cold Steel Tonsillectomy

**DOI:** 10.1155/2020/8060971

**Published:** 2020-05-18

**Authors:** Youssef El Sayed Ahmad, Jade Nehme, Nabil Moukarzel

**Affiliations:** ^1^Lebanese University, Baabda-Brazilia Street, Beirut, Lebanon; ^2^Otolaryngology Head and Neck surgery, Sacré-Coeur Hospital-Baabda, Lebanese University, Beirut, Lebanon; ^3^ENT-H&N Surgery, Sacré-Coeur Hospital, Lebanese University, Baabda-Brazilia Street, Beirut, Lebanon

## Abstract

**Objectives:**

Adenotonsillectomy (AT) is amongst the most widely performed pediatric surgeries in the United States (US) and the whole world. AT includes two major surgical techniques: total tonsillectomy (TT) and partial tonsillectomy (PT). Several studies have been conducted to evaluate the difference between TT and PT and assess the comparative effectiveness, benefits, and sequelae between both. In Lebanon, very few studies were done tackling this issue and assessing its sequelae on the pediatric population.

**Methods:**

A prospective study was conducted including pediatric patients aged between 2 and 9 years, who were admitted for partial tonsillectomy (PT) or total tonsillectomy (TT) in 2018. An estimated number of children included were 50: 25 patients underwent PT and 25 patients underwent TT. Patients were sent home on day 1 post-op with a questionnaire that evaluates the following over the first 10 days post-op: pain using the Wong–Baker Faces Pain Rating Scale and the “Parents Postoperative Pain Measure” (PPPM) questionnaire, and appetite using the visual analogue scale (VAS).

**Results:**

Patients in the PT group and in the TT group had no demographical differences in terms of age, BMI, exposure to smoking, area of living, and attending a day care center. Comparison between PT and TT revealed a significant difference in both pain and appetite scales. Patients who underwent PT had significantly lower PPPM scores on the 1^st^, 2^nd^, 4^th^, 5^th^, 6^th^, and 10^th^ day after surgery compared to the TT patients. Further validation was revealed by the Wong–Baker Faces Pain Rating Scale, showing that the PT surgery group experienced significantly less postoperative pain compared to the TT surgery group. Assessing the appetite using the visual analogue scale favored PT over TT. Comparisons revealed that most PT patients returned to their normal eating habits starting at day 4 while this was applicable in the TT group at day 10. Postoperative pain improved from day 1 to day 10 in both surgical groups.

**Conclusion:**

In conclusion, the recovery process after the PT surgery causes less postoperative morbidity, thus an earlier return to normal activity compared to the TT. The patients of the latter group are affected by more pain and less appetite over the first 10 days after the surgery.

## 1. Introduction

In the first century BC, Aulus Cornelius Celsus, a Roman physician, was the first to describe the surgical removal of tonsils by applying vinegar and milk to ensure hemostasis at the surgical site. Technique evolved and was refined with time till 1909 when Cohen adopted ligature of bleeding vessels to control perioperative bleeding, and tonsillectomy became a common and safe procedure [1].

Complications associated with total tonsillectomy, such as pain, bleeding, and eating difficulties, have led surgeons to consider partial tonsillectomy, which was introduced by Philip Physick using the tonsil guillotine during the procedure. This surgery was more common until 1930s, but was then replaced by total tonsillectomy for fear of regrowth and recurrence of symptoms and sometimes tonsillitis of the residual tonsillar tissue [2].

However, because of the mentioned complications post-total tonsillectomy, in addition to readmission sometimes due to dehydration or delayed bleeding, the concept of partial tonsillectomy came back again into mind. Proponents of this procedure believe that leaving a margin of tissue on the tonsillar capsule may speed healing and reduce inflammation, thus decreasing postoperative pain and bleeding.

A systematic review was performed recently in 2017 by Sathe et al. to compare the effectiveness between partial tonsillectomy and total tonsillectomy [3]. In this review, 6 studies were included to address the efficiency of PT vs. TT worldwide. Recurrent throat infection was a prominent indication for children to undergo TT than receiving PT, although differences were not statistically significant. Children undergoing PT vs. TT had consistently more favorable outcomes. Considering the quality of life, no significant difference was observed between patients undergoing partial tonsillectomy and total tonsillectomy regarding changes in physical suffering, sleep disturbances, speech issues, or caregiver concerns [3]. Yet PT was associated with a decrease in emotional distress and activity limitations [4], but accompanied with tonsillar regrowth (6%) and symptom recurrence compared to total tonsillectomy without statistically significant difference in the long run [3]. Note that children undergoing partial tonsillectomy returned to normal diet approximately 4 days sooner than children undergoing total tonsillectomy when comparing TT and PT cold techniques [3]. In a study by Wang et al., PT was more advantageous as it was followed by a lower hemorrhage rate compared to TT (risk ratio of 0.28 favoring tonsillotomy), shorter operation time, and faster pain relief. However, in long-term follow-up, no significant difference was noticed regarding the resolution of upper-airway obstructive symptoms, quality of life, or postoperative immune function [5]. On a side note, significant heterogeneity in study protocols and the variety of available surgical techniques made meta-analysis and interpretation of certain parameters such as operative time and quality of life largely unreliable in terms of determining the superiority of one technique to another.

In this study, we compared postoperative outcomes among children undergoing partial and total tonsillectomy taking into consideration the patient's and the parent's feedback.

## 2. Materials and Methods

This is a prospective study including pediatric patients aged between 2 and 9 years admitted for partial or total tonsillectomy during 2018. Exclusion criteria included mental retardation, known or suspected congenital or hereditary abnormalities, medical conditions associated with chronic pain such as sickle cell disease or musculoskeletal deformities, children with noncorrected significant visual impairment or hearing loss, children undergoing another procedure at the same time other than adenoidectomy and myringotomy with insertion of tubes, children of whom caregivers are unable to fill in the questionnaire (illiterate, visual disabilities, etc.), and revision surgeries.

PT was performed using the monopolar cautery and TT was performed using the traditional cold technique.

For the PT, the monopolar is set on 30 Watt of the desiccation (coagulation, pinpoint) mode and a needle tip is used. The goal of resection was to leave only a thin concave lymphoid remnant lying laterally of the plane of the anterior and posterior pillars [6].

For the TT, an incision is made on the anterior tonsillar pillar with a sickle knife, dissection continued with a Fisher tonsil knife in the correct plane and the inferior pole liberated using a tonsil snare. Hemostasis was performed using Vicryl 3-0 suture ties, and bipolar set on 20 Watt was used for minor mucosal bleed when necessary.

This study was conducted in a manner that warrants confidentiality of all included patients. Data collected had been deidentified prior to being stored at the principal investigator's office (YSA). Permission was granted by the institution's ethical committee before starting the study and the data collection.

Written informed consent was obtained from the parents of the patients for participation in this study.

Data collection included questionnaire surveys and forms that were developed exclusively for this study. Children enrolled in this study, who had been suffering from recurrent tonsillitis and/or obstructive sleep apnea (OSA), were operated for TT and PT.

Patients were sent home on day 1 postoperatively (post-op) with a questionnaire—to be answered by their parents—that evaluates the following:

- Postoperative pain using the Wong–Baker Faces Pain Rating Scale and the “Parents Postoperative Pain Measure” (PPPM) questionnaire.
- Appetite using the visual analogue scale standardized as a 10 cm line with the “no appetite” and “usual appetite” labels present at both extremes. Parents place a mark on the location that represents their child's appetite. The distance between this mark and the “no appetite point” is measured with a ruler. Less than 4 cm is placed in the “poor appetite” category, between 4 and 7 cm is placed in the “acceptable appetite” category, and above 7 cm is placed in the “good appetite” category.
- Another questionnaire was filled by the parents before the surgery. It covers multiple aspects of the child's personal history (onset and duration of symptoms), family history (tonsillectomy in parents or siblings), and environmental aspects (living in a rural or urban area, day care center, indoor smoking at home, and presence of pets).

After completing the data collection process, data were entered into a large Microsoft Excel spreadsheet previously designed especially for this study. After that, the data were transferred into the Statistical Package of Social Science (IBM SPSS, version 22) which was used for data cleaning and analyses.

## 3. Results

50 consecutive patients, distributed equally between both groups, met the inclusion criteria. Characteristics of the subjects are presented in Table 1. Regarding the cause of surgery, in the PT group, 80% of patients were operated for obstructive sleep apnea, 20% for recurrent tonsillitis, and 0% for simultaneous occurrence of both conditions, compared to 28%, 44%, and 28% in the same order for the TT group. All patients in both groups received adenoidectomy, while myringotomy was performed in 64% and 16% of cases in the PT and TT groups, respectively. The previous history of reflux was present in 16% of patients operated for PT and 12% of those operated for TT, and this difference is not statistically significant (*p*=0.684).

### 3.1. Pain Management

**Figure 1:**
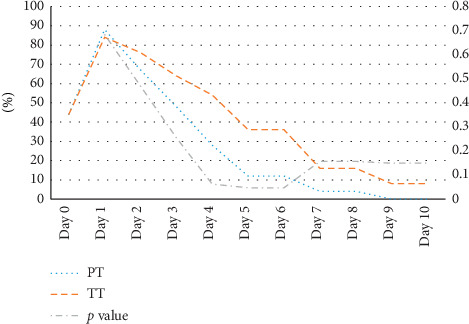
Percentage of patients receiving painkillers for partial tonsillectomy (PT) and total tonsillectomy (TT) surgery groups starting on the day of surgery till day 10. Fisher's exact test was used comparison.

Throughout the 10-day follow-up period, difference in the need for painkillers did not reach statistical significance between both groups except for days 5 and 6 (*p* value = 0.047) with a trend towards less need for pain management in the PT group. The painkillers varied between pills, syrup, or suppositories of acetaminophen (Figure 1).

### 3.2. Parents Postoperative Pain Measure

**Figure 2:**
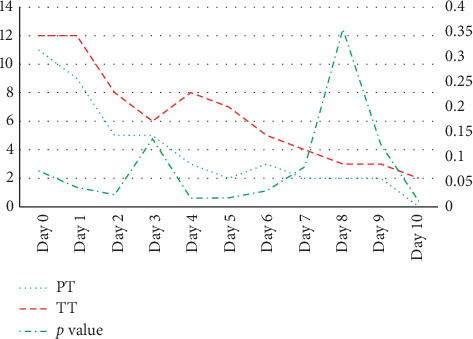
Parents Postoperative Pain Measure (PPPM) scores in the partial tonsillectomy (PT) and total tonsillectomy (TT) surgery groups starting on the day of surgery till day 10. Scores were reported in median (range). Mann–Whitney *U* test was used for comparison.

With a cutoff value of 6, more being significant pain and less nonsignificant, the TT children experienced significant pain until day 5, while the PT group pains lasted for 1 day only. The pain scores were significantly higher on days 1, 2, 4, 5, 6, and 10 in the TT surgery group compared to the PT group (Figure 2).

### 3.3. Wong–Baker Faces Pain Rating Scale

**Figure 3:**
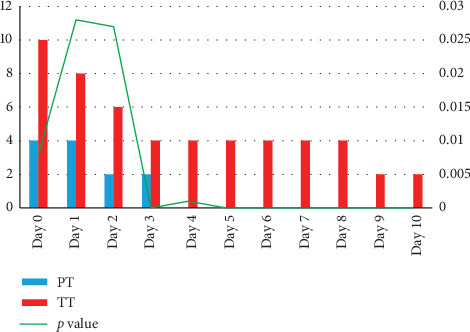
Wong–Baker Faces Pain Rating Scale scores in the partial tonsillectomy (PT) and total tonsillectomy (TT) groups starting on the day of surgery till day 10. Scores were reported in median (range). Mann–Whitney *U* test was used for comparison.

The pains score decreased slowly from 8 (0–10) at day 1 to 2 at days 9 and 10. The difference in pain score assessed by children was always significant between the PT and TT surgery groups during the follow-up period. This suggests that, according to children, the TT group experienced a higher postoperative pain compared to the PT surgery group (Figure 3).

### 3.4. Visual Analogue Scale for Appetite

**Figure 4:**
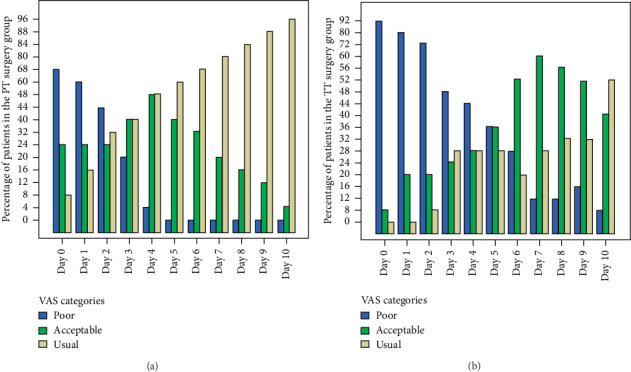
A bar graph of the percentage of patients in visual analogue scale (VAS) categories over the 10-day follow-up period in partial tonsillectomy (PT) (a) and total tonsillectomy (TT) (b) surgery groups.

On the first 4 days after surgery (from day 0 to day 3), no significant differences were observed in the PT vs. the TT surgery groups. The difference in the VAS score was highly significant on the 4^th^ follow-up day (*p* value = 0.004). The significant difference was sustained from day 4 till day 10, where a noticeable fast shift in the appetite was observed in the PT group vs a slow one in the TT group. On the last follow-up day, 24 (96%) in the PT group restored their usual appetite, whereas only 13 (52%) had reached this stage (*p* value = 0.002) (Figure 4).

## 4. Discussion

Tonsillectomy is still one of the most common procedures performed in the field of ear, nose, and Throat (ENT). It is considered as a minor surgery since patients are frequently discharged at the same day of the intervention, despite that they might potentially have a difficult postoperative course [7]. Partial tonsillectomy was more common until the 1930s, but was then replaced by total tonsillectomy, mostly for fear of regrowth. Surgeons, especially in the late 1980s, renewed interest in this surgery after realizing that total tonsillectomy was followed almost always by significant pain, decreased appetite, and sometimes bleed. Conventional (total) tonsillectomy involves the removal of the tonsillar capsule, while PT preserves the capsule by shaving away the tonsils using a variety of instruments [8].

In a study on 1,445 patients undergoing total tonsillectomy, hemorrhage rate was estimated to occur in 2.62% of the participants. When it comes to pain, it seems that it is an inescapable complication, which may be severe enough to restrict oral intake resulting in possible dehydration and hospitalization [9]. In another study conducted in Sweden comparing post-op recovery among children undergoing tonsillotomy or tonsillectomy, a significant difference was found concerning post-op pain and decreased appetite, with higher rates reported in the total tonsillectomy group [7]. In Lebanon, to the best of our knowledge, there is only one study comparing outcomes of PT and TT, using the microdebrider and electrocautery techniques, respectively [10]. Our study will be the first to compare the frequently used monopolar technique for PT and cold technique for TT.

In our study, patients were equally divided into two groups: the first representing children who underwent partial tonsillectomy and the second denoting total tonsillectomy. The mean age of patients of both groups was 4 years and the mean BMI was also similar (15.6 kg/m^2^). A statistically significant difference was found in genders of patients between PT and TT where those who underwent TT were mainly males (76%) whereas among the group of patients who undertook PT, females were slightly higher than males.

Considering the cause of surgery, obstructive sleep apnea was the main indication in the PT group of patients (80% of patients had obstructive sleep apnea versus 20% had recurrent infections), whereas recurring throat infections were the major cause of surgery in TT (44% of patients had recurrent infections, 28% had obstructive sleep apnea, and the rest had both). This is consistent with a similar study conducted by Wolpoe et al. where amongst 350 children who were suffering from obstructive sleep apnea, 234 children underwent partial tonsillectomy and 107 children underwent total tonsillectomy [11]. The authors concluded that partial tonsillectomy is safer and more reliable than total tonsillectomy for children with obstructive sleep apnea, as it prompts less postoperative pain, faster recovery, and better quality of life.

In our study, the median PPPM scores decreased gradually in both surgery groups from day 1 until day 10. Nevertheless, median PPPM score was significantly higher in TT than in PT on days 1, 2, 4, 5, 6, and 10. Consequently, more patients were given painkillers in the TT surgery group than in the PT group from day 2 until day 10, with a statistically significant difference between the groups on days 5 and 6. The Wong–Baker Faces Pain Rating Scale was also used in this study for further validation in assessing postoperative pain. Results were consistent with the PPPM scoring where TT patients always had higher pain scores than PT patients from day 1 until day 10, which was statistically significant.

In a review of literature for similar studies that assess pain in children undergoing tonsillectomy (partial vs. total) postoperatively, PT was almost always associated with less pain [7]. The rapidness in pain relief with partial tonsillectomy vs. total tonsillectomy was also reported by Wang et al. [5]. In another study conducted on 76 patients comparing the intraoperative and postoperative clinical results of bipolar electrocautery tonsillectomy and conventional tonsillectomy techniques in children with respiratory tract obstruction, children who underwent bipolar electrocautery tonsillectomy group had significantly less scores in pain throughout their recovery period, duration until resumption of oral intake, intake of painkiller, recovery time, and postoperative pain than those who had conventional cold tonsillectomy [12]. Besides, a study done by Sobol et al. comparing postoperative recovery after microdebrider intracapsular or monopolar electrocautery tonsillectomy showed no significant difference in the number of days taken for the resolution of pain or resumption of normal activity between the 2 groups, with the resumption of near-normal dietary intake being achieved 1.7 days earlier in patients receiving microdebrider intracapsular tonsillectomy compared with monopolar electrocautery tonsillectomy [13].

Regarding the second postoperative parameter, visual analogue scale was used to access the appetite of the patients. In our study, our results revealed that there was a significant difference in the appetite between PT and TT starting at day 4. After this cutoff point, most of the patients in the PT group developed an acceptable or usual appetite whereas patients in the TT still recorded a considerable percentage in the poor appetite category.

Our results are in concordance with what was previously reported in the literature. A recent study in Sweden revealed that PT patients return to normal eating habits 2 days earlier than the TT patients [14]. Additionally, these results are equivalent to the results of a systematic review of randomized clinical trials by Walton et al. comparing PT and TT [15]. These studies revealed that PT demonstrates less morbidity compared to the TT surgery group with a shorter period of pain treatment, faster healing process, and faster return to regular eating habits [14, 15]. Importantly, a suitable postoperative oral intake of food and drinks after both TT and PT is crucial as it promotes child's recovery. Dehydration, for example, leads to a vicious cycle of less drinking, then less food intake, and accordingly an increase in morbidity (pain, bleeding, and infections) [7].

This study has a number of limitations. First, not all potential postoperative complications associated with tonsillectomy were studied since the follow-up time chosen was 10 days (short-term complications only included). Second, the sample size in our study was relatively small which made the analysis of bleeding rates statistically not valid. We tried to mitigate these problems by using patient and family grading scales in addition to a daily filled questionnaire for 10 days.

## 5. Conclusion

In conclusion, the recovery process after the PT surgery causes less postoperative morbidity. The patients of the latter group are affected by less pain over the first 10 days after the surgery and returned faster to usual appetite starting at the 4^th^ postoperative day.

## Figures and Tables

**Table 1:** Demographic characteristics of the partial tonsillectomy (PT) and total tonsillectomy (TT) groups.

	PT	TT	*p* value
	*n* = 25	*n* = 25	
BMI (kg/m^2^)	15.6 (13.3–23.9)	15.6 (14.5–18.1)	0.586
Age	4 (2–7)	4 (2–9)	0.789
Gender			
Male	12 (48%)	19 (76%)	0.041
Female	13 (52%)	6 (24%)	
Second-hand smoking			
Yes	7 (28%)	12 (48%)	0.145
Area of living			
Rural	12 (48%)	14 (56%)	0.571
Urban	13 (52%)	11 (44%)	
Day care center			
Yes	5 (20%)	1 (4%)	0.082

Fisher's exact test was used for dichotomous variables, and Mann–Whitney *U* test was used for comparison of continuous variables between the two groups.

## Data Availability

The data used to support the findings of this study are available from the corresponding author upon request (youssefelsayedahmad@gmail.com).
